# Association of Opioid Use With Pain and Satisfaction After Dental Extraction

**DOI:** 10.1001/jamanetworkopen.2020.0901

**Published:** 2020-03-13

**Authors:** Romesh P. Nalliah, Kenneth R. Sloss, Brooke C. Kenney, Sarah K. Bettag, Shernel Thomas, Kendall Dubois, Jennifer F. Waljee, Chad M. Brummett

**Affiliations:** 1 University of Michigan School of Dentistry, Ann Arbor; 2 Michigan Opioid Prescribing Engagement Network (Michigan OPEN), Ann Arbor; 3 Department of Surgery, University of Michigan Medical School, Ann Arbor; 4 Division of Pain Medicine, Department of Anesthesiology, University of Michigan Medical School, Ann Arbor

## Abstract

**Importance:**

Dentists commonly prescribe opioids to relieve pain after tooth extraction. Understanding the differences in patient-reported outcomes between opioid users and nonusers could encourage the adoption of more conservative and appropriate prescribing practices in dental medicine.

**Objective:**

To evaluate whether pain and satisfaction scores reported by patients who used opioids after tooth extraction were similar to the levels reported by patients with no opioid use.

**Design, Setting, and Participants:**

This quality improvement study was conducted in the 14 dental clinics of the University of Michigan School of Dentistry. Eligible adult patients of these clinics who underwent routine or surgical extractions between June 1, 2017, and December 31, 2017, were contacted by telephone within 6 months of the procedure. Patients were surveyed about the type of extraction, use of prescription opioid (if given), use of nonopioid analgesics, pain levels, and satisfaction with care after the procedure. Data analysis was conducted from February 1, 2018, to July 31, 2018.

**Main Outcomes and Measures:**

The primary outcome was self-reported pain as assessed by the question, “Thinking back, how would you rate your pain in the first week after your dental procedure?” with a 4-point pain scale of no pain, minimal pain, moderate pain, or severe pain. Secondary outcomes included self-reported satisfaction with care as assessed by a Likert scale ranging from 1 to 10, in which 1 was extremely dissatisfied and 10 was extremely satisfied.

**Results:**

The final cohort comprised 329 patients, of whom 155 (47.1%) underwent surgical extraction (mean [SD] age, 41.8 [18.1] years; 80 [51.6%] were men) and 174 (52.9%) underwent routine extraction (mean [SD] age, 52.4 [17.9] years; 79 [45.4%] were men). Eighty patients (51.6%) with surgical extraction and 68 (39.1%) with routine extraction used opioids after their procedure. In both extraction groups, patients who used opioids reported higher levels of pain compared with those who did not use opioids (surgical extraction group: 51 [63.8%] vs 34 [45.3%], *P* < .001; routine extraction group: 44 [64.7%] vs 35 [33.0%], *P* < .001). No statistically significant difference in satisfaction was found between groups after surgical extraction (median [interquartile range] scores: 9 [7-10] for nonopioid group vs 9 [8-10] for opioid group) and routine extraction (median [interquartile range] scores: 10 [8-10] for nonopioid group vs 9 [7-10] for opioid group).

**Conclusions and Relevance:**

This study found that patients who used opioids after tooth extraction reported significantly higher levels of pain compared with nonusers, but no difference in satisfaction was observed.

## Key Points

**Question:**

Are patient-reported pain and satisfaction scores similar between opioid users and nonopioid users after routine or surgical dental extractions?

**Findings:**

In this quality improvement study of 329 patients who underwent either surgical or routine extractions, patients who used opioids after the procedure reported higher levels of pain compared with nonopioid users. Those who did not use opioids reported similar satisfaction levels as the opioid users.

**Meaning:**

These findings suggest that nonopioid analgesics, instead of opioids, should be the first-line medication for dental extraction.

## Teaser

This quality improvement study compares patient-reported pain and satisfaction scores within 6 months of a tooth extraction among patients who used vs did not use opioids.

## Introduction

In 2017, the number of opioid-related deaths in the United States reached 49 068, an approximately 16% increase from the 2016 rate.^1^ The opioid crisis is the only public health issue in the United States with worsening associated mortality. Largely owing to opioid-related overdoses, life expectancy in the United States dropped for the third consecutive year since World War I.^2^

The Centers for Disease Control and Prevention and other national efforts have largely focused on the prescribing practices of primary care and pain physicians^3,4^; however, dentists and oral surgeons have received more attention in recent years. Between 2010 and 2016, new dental procedure–associated opioid prescriptions to opioid-naive adult patients increased by 68%.^5^ Furthermore, dentists are among the most common prescribers for minor patients, and for many individuals, dental opioid prescriptions represent their first exposure to opioids.^6^ Previous studies of surgical cohorts have shown that opioid prescribing after a surgical procedure is not associated with patient satisfaction or the likelihood for refill requests.^7^ However, recent research has identified, for the first time, that dentists have an important role in the transition of opioid-naive users to persistent opioid users after a wisdom tooth extraction.^7,8,9^

Although a growing body of data is available that demonstrates that meaningful reduction, or even elimination, of opioids after a surgical procedure is appropriate,^9,10,11^ dental and oral surgical data are lacking. Randomized clinical trials in dentistry have shown that acetaminophen and nonsteroidal anti-inflammatory drugs (NSAIDs) are equivalent or even superior to opioids for dental pain.^12^ However, concerns remain from the dental community about the clinical relevance of the selected participants in these trials, and opioid prescribing after extractions remains common.^13^ Currently, knowledge is scarce about patient use (or nonuse) of the opioids prescribed by dentists. This quality improvement study was designed to test the hypothesis that patients who did not use opioids after routine or surgical dental extraction would report similar pain and satisfaction scores as patients who used opioids.

## Methods

The study protocol was not considered to be regulated quality improvement research by the institutional review board of the University of Michigan, Ann Arbor; therefore, informed consent was not obtained. The study followed the Strengthening the Reporting of Observational Studies in Epidemiology (STROBE) reporting guideline.^14^

We identified eligible local patients (in Ann Arbor, Michigan) by searching the electronic dental records at the University of Michigan School of Dentistry, the first public university–affiliated dental school in the United States. The school was the ideal site for a study of opioid prescribing. Not only does it serve a large patient population, but it also has dental students, hygiene students, dental residents, and a private practice in which the dental faculty members treat patients. This study was conducted in the 14 clinics run by the University of Michigan School of Dentistry.

### Study Cohort

Patients were surveyed if they had undergone a routine or surgical extraction procedure at the University of Michigan School of Dentistry between June 1, 2017, and December 31, 2017. *Routine extraction* was defined as requiring no conjunctive removal of bone or extraction of soft tissue because the teeth were visible and above the gum line. *Surgical extraction* was defined as requiring an incision into the connective tissue to gain access to the tooth. Following a similar structure used in previous surgical quality improvement studies,^9,15^ we contacted patients by telephone within 6 months after their procedure. We asked them whether they received an opioid prescription and, if so, to describe the number of opioid pills they used, the instructions for opioid consumption they followed, their opioid storage practices, their use of nonopioid analgesics, their pain before and after the procedure, and their satisfaction with their pain management. A 6-month postextraction window was used to ensure an adequate sample size while maintaining a time frame in which patients could recall their opioid use and patient experience. This process aligned with previous quality improvement efforts for surgical opioid prescribing as well as for perioperative pain and satisfaction.^9,15,16^ Exclusion criteria were patients who reported use of opioids before their procedure, an opioid fill that was not consistent with the dental record, extractions outside of the study time frame, and being younger than 18 years.

### Outcomes

The primary outcome was self-reported pain as assessed by the question, “Thinking back, how would you rate your pain in the first week after your dental procedure?” with a 4-point pain scale of no pain, minimal pain, moderate pain, or severe pain. Secondary outcomes included self-reported satisfaction with care as assessed by a Likert scale ranging from 1 to 10, in which 1 was extremely dissatisfied and 10 was extremely satisfied. For the cohort who received opioid prescriptions, patient-reported opioid consumption was converted into oral morphine equivalents (OMEs) and standardized to 5-mg–equivalent hydrocodone bitartrate pills.

### Statistical Analysis

Both routine extraction and surgical extraction were each selected by a sufficient sample size of surveyed respondents and were evaluated separately. Both cohorts had patients who received an opioid prescription but did not fill the prescription and thus were considered nonopioid users. Descriptive statistics were calculated for patient demographic characteristics and postprocedure behavior. Univariate differences between patients who used an opioid (opioid group) and those who did not (nonopioid group) were assessed with χ^2^ tests or unpaired, 2-tailed *t* tests. Among those who received an opioid prescription, the distributions of OMEs prescribed and OMEs consumed were compared using the Wilcoxon signed-rank test. Differences in pain level and satisfaction with pain management among those who used an opioid and those who did not were compared using the Wilcoxon Mann-Whitney test. In addition, we assessed for differences in these outcomes after stratifying patients by age group (18-34, 35-64, 65-74, and ≥75 years) and sex. The primary outcome compared those who reported any postprocedural opioid use with those who did not report use (0 pills) regardless of whether they received an opioid prescription.

All analyses were conducted with SAS, version 9.4 (SAS Institute Inc). Two-sided *P* < .05 was considered statistically significant. Because we did not have available data for postextraction pain before the study, a formal power analysis was not conducted. Data analysis was conducted from February 1, 2018, to July 31, 2018.

## Results

**Figure 1.**
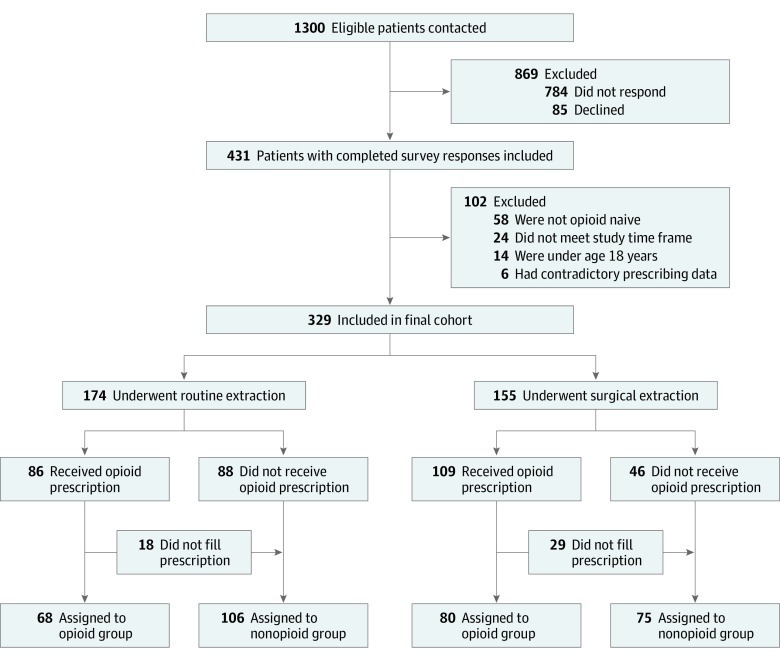
Patient Flow Diagram Of the 1300 eligible patients, 784 could not be contacted and 85 refused to participate. An additional 102 patients were excluded for reasons such as being younger than 18 years, having the procedure or response outside of the study period, and using preoperative opioids. The final cohort comprised 329 patients assigned to the opioid and nonopioid groups. Sensitivity analyses were conducted in which these patients were assigned to the opioid group and another set of analyses in which they were excluded.

The patient flow diagram is shown in Figure 1. The final cohort consisted of 329 patients, including 155 (47.1%) who underwent surgical extractions (mean [SD] age, 41.8 [18.1] years; 80 [51.6%] were men) and 174 (52.9%) who underwent routine extractions (mean [SD] age, 52.4 [17.9] years; 79 [45.4%] were men). Among patients with surgical extraction, 80 (51.6%) used opioids after their procedure, whereas 68 patients (39.1%) with routine extraction used opioids. Baseline characteristics showed that the opioid users were considerably younger and comprised a higher percentage of female patients, but the differences were not statistically significant (Table).

**Table.** Baseline Patient Characteristics and Postprocedural Behavior Among Dental Cohort Stratified by Procedure and Postprocedural Opioid Use

Variable	Surgical Dental Extraction				Routine Dental Extraction			
	No. (%)			*P* Value	No. (%)			*P* Value
	Nonopioid Group (n = 75 [48.4%])	Opioid Group (n = 80 [51.6%])	Total (n = 155)		Nonopioid Group (n = 106 [60.9%])	Opioid Group (n = 68 [39.1%])	Total (n = 174)	
Patient characteristics								
Male	44 (58.7)	36 (45.0)	80 (51.6)	.09	54 (50.9)	25 (36.8)	79 (45.4)	.07
Age, mean (SD), y	46.3 (19.5)	37.5 (15.8)	41.8 (18.1)	.003	56 (17.9)	46.9 (16.8)	52.4 (17.9)	.001
Consumes alcoholic drinks	28 (37.3)	28 (35.0)	56 (36.1)	.76	29 (27.4)	20 (29.4)	49 (28.2)	.77
Uses or has used recreational drugs	8 (10.7)	7 (8.8)	15 (9.7)	.69	6 (5.7)	6 (8.8)	12 (6.9)	.42
Has had a problem with alcohol and/or drugs	3 (4.0)	6 (7.5)	9 (5.8)	.50	2 (1.9)	1 (1.5)	3 (1.7)	>.99
Postprocedural behavior								
Used medications to treat pain (eg, ibuprofen, celecoxib, or naproxen sodium) after leaving the dental clinic	46 (61.3)	46 (57.5)	92 (59.4)	.63	49 (46.2)	32 (47.1)	81 (46.6)	.91
Used acetaminophen or NSAIDs to treat pain after leaving the dental clinic	25 (33.3)	18 (22.5)	43 (27.7)	.13	27 (25.5)	20 (29.4)	47 (27.0)	.57
Called or visited the dental clinic with any pain concerns associated with dental procedure	10 (13.3)	9 (11.2)	19 (12.3)	.69	10 (9.4)	12 (17.7)	22 (12.6)	.11
Took any other opioid pain medication in the month after dental procedure	4 (5.3)	3 (3.8)	7 (4.5)	.71	8 (7.6)	3 (4.4)	11 (6.3)	.41

Abbreviation: NSAIDs, nonsteroidal anti-inflammatory drugs.

### Pain in the Opioid Group

**Figure 2.**
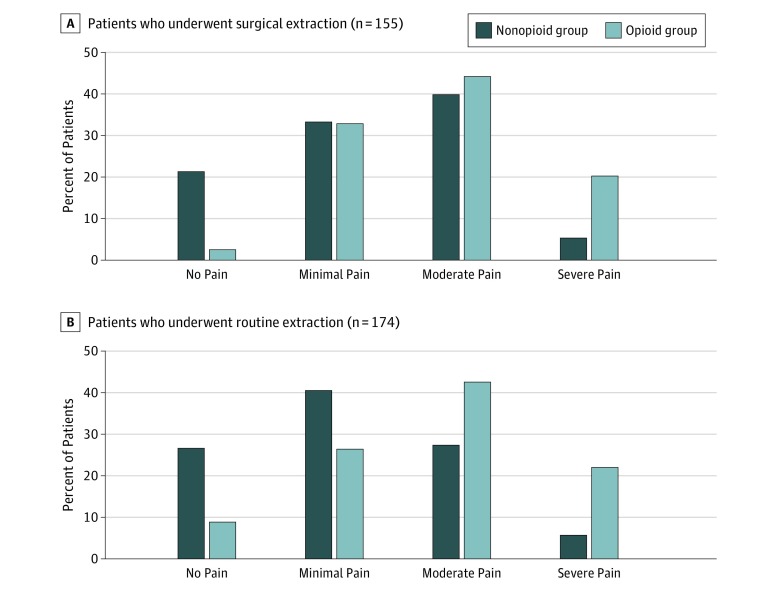
Pain Level of Opioid Users After Dental Procedure Patients who used opioids reported worse pain compared with nonusers. A, Fifty-one patients (63.8%) who used opioids during surgical dental extraction reported moderate to severe pain, whereas only 34 patients (45.3%) who did not use opioids reported moderate to severe pain. B, Forty-four patients (64.7%) who used opioids during routine dental extraction reported moderate to severe pain, whereas only 35 patients (33.0%) who did not use opioids reported moderate to severe pain.

In both the surgical and routine extraction groups, patients in the opioid group reported worse pain compared with those in the nonopioid group (surgical extraction group: 51 [63.8%] vs 34 [45.3%], *P* < .001; routine extraction group: 44 [64.7%] vs 35 [33.0%], *P* < .001) (Figure 2). The opioid group had more patients reporting moderate to severe pain for both routine and surgical extractions. Analysis of this 4-point ordinal pain scale produced a median (interquartile range [IQR]) of 3 (2-3) for the surgical extraction opioid group and 2 (2-3) for the nonopioid group (*P* < .001). The routine extraction opioid group had a median (IQR) of 3 (1-3) compared with the nonopioid group median (IQR) of 2 (1-3) (*P* < .001). The percentage of patients in the routine extraction opioid group who reported pain concerns was higher than those in the nonopioid group, but the difference was not statistically significant (Table). Likewise, no statistically significant differences in the use of nonopioid analgesics (NSAIDs or acetaminophen) were found between opioid and nonopioid users in the surgical extraction and routine extraction groups. We did not observe statistically significant differences in pain score when stratified by sex or age group in either extraction group.

### Satisfaction Between the Opioid and Nonopioid Groups

**Figure 3.**
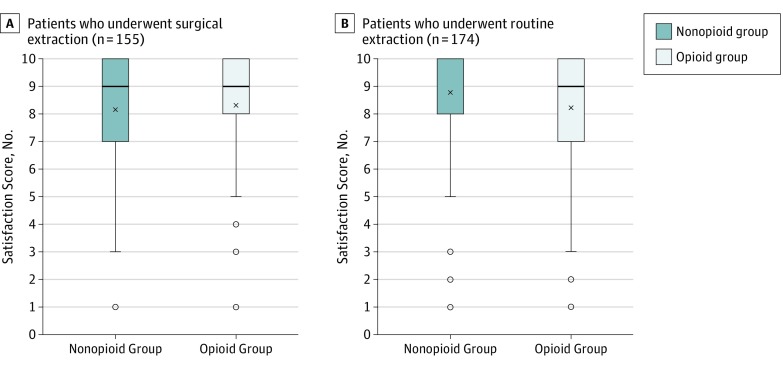
Satisfaction of Both Opioid and Nonopioid Users After Dental Procedure A and B, Patient-reported satisfaction was high for those who underwent either surgical or routine dental extraction. No statistically significant difference in overall satisfaction with pain management was found between opioid users and nonusers. Satisfaction scores were measured with a Likert scale ranging from 1 to 10, with 1 as extremely dissatisfied and 10 as extremely satisfied. The median satisfaction score was 10 in the nonopioid group of patients who underwent routine extraction; therefore, the top of box overlaps with the median. Bottom whisker indicates the local minimum; bottom line of the box, first quartile, and top line, third quartile; X, the mean, and midline inside the box, the median; and dots below the bottom whisker, outliers or more than 1.5 times the length of the interquartile range.

Satisfaction was high in both routine and surgical extraction groups. No differences in satisfaction scores were found between the nonopioid group and opioid group after surgical extraction (median [IQR] scores: 9 [7-10] vs 9 [8-10]) and after routine extraction (median [IQR] scores: 10 [8-10] vs 9 [7-10]) (Figure 3), nor were differences observed when stratified by sex and age group. The median (IQR) data of satisfaction levels are provided in eTable 1 in the Supplement.

### Opioid Use Among Patients Receiving Prescriptions

**Figure 4.**
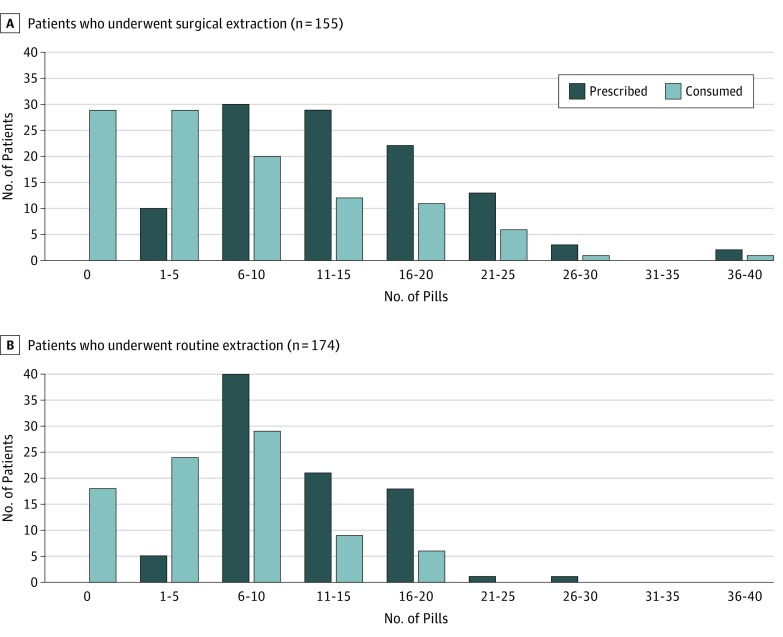
Opioid Prescription vs Consumption After Dental Procedure A, Among patients who underwent surgical dental extraction, the median (interquartile range [IQR]) number of pills prescribed, standardized to hydrocodone bitartrate, 5 mg, was 12 (8-18.9), with a median (IQR) oral morphine equivalent (OME) of 60 (40-94.5). Consumption of these opioids was significantly less, with a median (IQR) number of 5 (0-12) pills and a median (IQR) OME of 25 (0-60) (*P* < .001). The total excess prescribing among patients who had a surgical extraction was 683 pills. B, Among patients who underwent routine dental extraction, the median (IQR) number of pills prescribed was 10 (8-15) and the median (IQR) number of pills consumed was 5.7 (1.8-9), with a median (IQR) OME of 28.5 (9-45) (*P* < .001). The total excess prescribing among patients who had a routine extraction was 463 pills.

In total, 109 patients (70.3%) who underwent surgical extraction and 86 patients (49.4%) who underwent routine extraction received an opioid prescription after their procedure. Among those in the surgical extraction group, the median (IQR) OME prescribed was 60 (40-94.5). When standardized to hydrocodone, 5 mg, the median (IQR) was 12 (8-18.9) pills. Consumption of these opioids was significantly less, with a median (IQR) OME of 25 (0-60) with a median (IQR) number of pills consumed of 5 (0-12) (*P* < .001) (Figure 4A). Among patients in the routine extraction group, the median (IQR) OME was 50 (40-75) with a median (IQR) number of pills prescribed of 10 (8-15). Median (IQR) consumption was an OME of 28.5 (9-45) and a median (IQR) number of pills consumed of 5.7 (1.8-9) (*P* < .001) (Figure 4B). The total excess prescribing among the 195 total surgical and routine extractions was 1146 pills.

### Additional Analgesic Use and Telephone Calls Regarding Pain 

No statistically significant differences in the number of patients who used NSAIDs or acetaminophen were found between the opioid group and nonopioid group for both routine extractions (20 [29.4%] vs 27 [25.5%]; *P* = .57) and surgical extractions (18 [22.5%] vs 25 [33.3%]; *P* = .13) (Table). A small percentage of patients in both opioid and nonopioid groups reported using other opioid medications in the month after the extraction, but this percentage did not differ between the surgical procedure (3 [3.8%] vs 4 [5.3%]; *P* = .71) and the routine procedure (3 [4.4%] vs 8 [7.6%]; *P* = .41) cohorts. Approximately 12% of patients in both the routine and surgical extraction cohorts reported contacting the dental clinic for pain associated with the extraction. Although a higher percentage of patients in the routine extraction opioid group reported making telephone calls compared with the nonopioid group, this difference was not significant (12 [17.7%] vs 10 [9.4%]; *P* = .11). Rates of telephone calls were similar in the surgical extraction cohort (9 [11.2%] vs 10 [13.3%]; *P* = .69).

### Sensitivity Analyses

Patients who received opioid prescriptions but did not fill them were regrouped for 2 sensitivity analyses. First, in an intent-to-treat analysis, these patients were categorized in the opioid group. In the second sensitivity analysis, these patients were omitted from the analysis. For both sensitivity analyses, the self-reported moderate to severe pain levels and satisfaction outcomes were similar to those in the primary analyses above. Again, the opioid group compared with the nonopioid group reported greater pain first sensitivity analysis of pain in the surgical extraction group [65 (60.2%) vs 20 (43.5%), *P* = .03] and routine extraction group [51 (59.3%) vs 28 (31.9%), *P* < .001]; second sensitivity analysis of pain in the surgical extraction group [51 (64.6%) vs 20 (43.5%), *P* = .003] and routine extraction group [44 (64.8%) vs 28 (31.9%), *P* < .001]). Likewise, no differences in median (IQR) satisfaction levels were observed between the 2 groups (first sensitivity analysis of satisfaction in the surgical extraction group [9 (8-10) vs 9 (7-10), *P* = .66] and routine extraction group [9 (7-10) vs 10 (8-10), *P* = .17); second sensitivity analysis of satisfaction in the surgical extraction group [9 (7.5-10) vs 10 (8-10), *P* = .35] and routine extraction group [9 (7-10) vs 10 (8-10), *P* = .32]) (eTable 2 and eTable 3 in the Supplement).

## Discussion

In this quality improvement study, patients who underwent routine or surgical extractions reported more pain (Figure 2) and similar levels of patient satisfaction across the opioid group compared with the nonopioid group (Figure 3). Opioids have been used for decades to manage postoperative pain associated with dental extractions. However, evidence has suggested that 400 mg of ibuprofen and 1000 mg of acetaminophen taken concurrently may be more advantageous than any opioid-containing medication for dental pain.^12^ Moreover, opioids have been associated with nausea, vomiting, and constipation, and recent studies have demonstrated that use of an opioid after a wisdom tooth extraction was independently associated with new chronic opioid use.^13,17^ Although randomized clinical trials generally recruit well-selected cohorts who may not represent the patients seen in clinical practice, data from these trials generally demonstrate the lack of effectiveness of opioids. These data also suggest that opioid prescribing is not needed to achieve high patient satisfaction. Although we do not suggest the routine prescribing of opioids for dental extractions, we acknowledge the rare cases in which opioid use is warranted.

Previous studies have demonstrated that patient factors, like centralized pain and anxiety, are associated with increased pain and opioid consumption after a surgical procedure and with poor opioid efficacy.^18,19,20^ Furthermore, some genes have been implicated in opioid responsiveness, including the *OPRM1* gene.^21,22^ These patient covariates were not measured in the present study and may account for some of the differences in the cohorts.

### Satisfaction Between the Opioid and Nonopioid Groups

Patient satisfaction with dental care is a complex metric given that the dentist’s technical competence is difficult for a patient to recognize.^23^ Research has demonstrated that convenience of a dental practice,^24^ cost of care,^25^ and even facility factors (such as availability of current magazines)^26^ seem to have implications for patient satisfaction. Dentists are health care professionals; however, most dentists must also run a small business, that is, their practice. More than 50% of dentists still run a solo practice^27^ and only approximately 3% of practices have 20 or more dentists.^28^ Any small business must optimize client satisfaction to protect and grow its brand; therefore, dentists must be cognizant of patient satisfaction. The current study showed that satisfaction was high in both routine and surgical extraction groups for opioid and nonopioid users. In many ways, this finding was not surprising given that multiple studies showed that interpersonal factors were the most important factors in patient satisfaction.^29,30,31^ Evidence has suggested that historical measures of technical skill (such as, “Was the dentist thorough in what they did?,” the answer to which indicated whether patients valued interpersonal factors more than actual patient outcomes) are more associated with interpersonal factors than with skill.^32^ Evidence has also shown that communication about cost is more important in satisfaction than the cost itself.^33^

### Pain Following Routine and Surgical Extraction

The current study demonstrated that patients who used opioids actually reported worse pain compared with those who did not in both surgical and routine extraction groups. However, the decision to prescribe an opioid is not based on universally recognized standards of practice in dentistry, and thus individual clinician-level variability in prescribing exists. In addition, patient pain perception is highly variable,^34,35^ and it is possible that this finding is associated with the intermingling of these highly variable factors rather than a true difference in analgesia effectiveness of opioids. Thoughtful preoperative discussion between clinician and patient about reasonable expectations may reduce the need for an opioid prescription after an extraction. Much is known about pain after dental extractions, and knowledge of pain intensity and duration can help the patient to feel more prepared to endure the discomfort for a limited time without opioids. The rates of acetaminophen and NSAIDs use were similar between the opioid group and nonopioid group, a finding that presents a quality improvement and patient education opportunity (the former group may not need an opioid prescription). Compelling evidence is also mounting for nonpharmacological methods for managing dental pain, such as acupuncture^36^ and behavioral techniques.

### Postprocedural Phone Calls 

No significant difference in the number of calls to the dental clinic about extraction-associated pain was found between the opioid group and the nonopioid group. For a small office that performs a high volume of extractions, this concern may lead to reducing opioid prescribing given that an increase in the number of calls and pain concerns could overburden the staff and the dentist and could serve as a barrier to practicing more conservative opioid prescribing; however, no such difference was revealed in this study. Previous studies of surgical cohorts demonstrated a lack of association between the amount of opioid prescribed and refills.^9,37^

### Unused Opioid Medications 

Previous research involving 79 participants demonstrated that approximately 54% of opioids prescribed by dentists for surgical procedures went unused.^38^ The small cohort in that study generated 1010 unused opioid pills. In the United States, no regulation has been established that requires dentists to educate their patients on the safe storage and disposal of their opioids. Unfortunately, unused drugs can enter a drug reservoir and play a role in opioid dependence, addiction, and mortality. In the current study, we found that 1146 pills went unused among 195 patients who underwent extractions.

Despite the potential for unmeasured confounding variables that can affect opioid prescribing, we recommend that, when considering these findings together with those of previous studies (which reported a lack of efficacy for opioids after dental extraction^12^ and which demonstrated morbidity after dental prescribing^13,17^), nonopioid analgesics be prescribed and used instead of opioids after dental extractions for most patients. Future studies may better identify those patients who are at risk for severe pain and ascertain whether opioids are efficacious in that cohort.

### Limitations

This study has several limitations that should be considered when reviewing the findings. First, this study had a retrospective design, which required participants to recall their opioid use, patient satisfaction, and pain perception regarding their past dental experience. The potential recall bias could be greater in patients who completed the survey 6 months after the extraction compared with those with a shorter window between the extraction and the survey. This potential bias would, however, be expected in both the opioid users and nonusers. Second, opioid prescribing was not randomized, and potential unmeasured confounding of patient and clinician characteristics could affect opioid prescribing and use. Third, the data were from a single academic medical center and may not be generalizable to other institutions or clinics. Although the total number of patients included was large for a study with patient-reported outcomes, we were unable to reach many patients, and a small number refused to participate. We did not find a statistical difference in sex between survey respondents and nonrespondents. A statistical difference in age was found, with respondents being approximately 5 years older on average. The surgical extraction group also had a substantially lower number of respondents who received an opioid prescription compared with nonrespondents.

## Conclusions

This study found that after routine or surgical extractions, patients who used opioids reported greater pain compared with those who did not use opioids. Moreover, opioid use was not associated with differences in patient satisfaction or telephone calls to report concerns about extraction-associated pain. We recommend the use of nonopioid analgesics instead of opioids for most patients undergoing dental procedures.

## Notes

Supplement. **eTable 1.** Pain and Satisfaction Outcomes (Primary Analyses) **eTable 2.** Sensitivity Analysis #1 (Intent to Treat): Patient Outcomes Among Dental Cohort Stratified by Procedure and Opioid Prescription Status **eTable 3.** Sensitivity Analysis #2 (Excluding Patients Who Were Prescribed But Did Not Fill or Consume Opioid): Patient Outcomes Among Patients Who Were Prescribed and Consumed Opioid and Those Patients Who Were Not Prescribed an Opioid, Stratified by Procedure
