# Error in Abstract, Results, and Figure 2 Caption

**DOI:** 10.1001/jamanetworkopen.2020.4463

**Published:** 2020-04-06

In the Original Investigation titled “Association of Opioid Use With Pain and Satisfaction After Dental Extraction,”^1^ published March 13, 2020, there were minor errors in the percentages of patients who reported higher levels of pain after dental extraction in those who used opioids compared with those who did not. These percentages should have appeared as 51 patients (64.6%) vs 34 (45.3%) in the surgical extraction group and 44 (64.8%) vs 35 (33.1%) in the routine extraction group. This article has been corrected.^1^
