# Supplementary material for: Association of Opioid Use With Pain and Satisfaction After Dental Extraction
Source: JAMA Netw Open. 2020 Mar 13;3(3):e200901. doi: 10.1001/jamanetworkopen.2020.0901 (PMC7070233; doi:10.1001/jamanetworkopen.2020.0901)
Supplement: Supplement. — eTable 1. Pain and Satisfaction Outcomes (Primary Analyses) eTable 2. Sensitivity Analysis #1 (Intent to Treat): Patient Outcomes Among Dental Cohort Stratified by Procedure and Opioid Prescription Status eTable 3. Sensitivity Analysis #2 (Excluding Patients Who Were Prescribed But Did Not Fill or Consume Opioid): Patient Outcomes Among Patients Who Were Prescribed and Consumed Opioid and Those Patients Who Were Not Prescribed an Opioid, Stratified by Procedure [file jamanetwopen-3-e200901-s001.pdf]

Supplementary Online Content

Nalliah RP, Sloss KR, Kenney BC, et al. Association of opioid use with pain and satisfaction after dental extraction. *JAMA Netw Open.* 2020;3(3):e200901. doi:10.1001/jamanetworkopen.2020.0901

eTable 1. Pain and Satisfaction Outcomes (Primary Analyses)

eTable 2. Sensitivity Analysis #1 (Intent to Treat): Patient Outcomes Among Dental Cohort Stratified by Procedure and Opioid Prescription Status

eTable 3. Sensitivity Analysis #2 (Excluding Patients Who Were Prescribed But Did Not Fill or Consume Opioid): Patient Outcomes Among Patients Who Were Prescribed and Consumed Opioid and Those Patients Who Were Not Prescribed an Opioid, Stratified by Procedure

This supplementary material has been provided by the authors to give readers additional information about their work.

©2020 Nalliah RP et al. *JAMA Network Open*

eTable 1. Pain and Satisfaction Outcomes (Primary Analyses)

	**Surgical Extraction**				**Routine Extraction**			
	**Non-Opioid Group N=75 (48.4)**	**Opioid Group N=80 (51.6)**	**Total (N=155)**	**P-value**	**Non-Opioid Group N=106 (60.9)**	**Opioid Group N=68 (39.1)**	**Total (N=174)**	**P-value**
**Outcomes.** Pain rating first week after dental procedure (n [%])[1]								
**1) No pain**	16 (21.3)	2 (2.5)	18 (11.7)	<0.01	28 (26.7)	6 (8.8)	34 (22.7)	<0.01
**2) Minimal pain**	25 (33.3)	26 (32.9)	51 (33.1)		43 (40.6)	18 (26.5)	61 (39.6)	
**3) Moderate pain**	30 (40)	35 (44.3)	65 (42.2)		29 (27.4)	29 (42.7)	58 (37.7)	
**4) Severe pain**	4 (5.3)	16 (20.3)	20 (13)		6 (5.7)	15 (22.1)	21 (13.6)	
**Overall satisfaction with pain management (1 = extremely dissatisfied, 10 = extremely satisfied); Median (IQR)[2]**	9 (7-10)	9 (8-10)	9 (8-10)	0.66	10 (8-10)	9 (7-10)	10 (8-10)	0.17

[1]One patient did not respond to pain score in the surgical extraction (opioid use) group
[2]Two patients did not respond to satisfaction in the surgical extraction group, one in opioid use and one in no opioid use

eTable 2. Sensitivity Analysis #1 (Intent to Treat): Patient Outcomes Among Dental Cohort Stratified by Procedure and Opioid Prescription Status

	**Surgical Extraction**				**Routine Extraction**			
	**Non-Opioid Group N=46 (29.7)**	**Opioid Group N=109 (70.3)**	**Total N=155 (100)**	**P-value**	**Non-Opioid Group N=88 (50.5)**	**Opioid Group N=86 (49.5)**	**Total N=174 (100)**	**P-value**
**Outcomes.** Pain rating first week after dental procedure (n [%])[1]								
**1) No pain**	9 (19.6)	9 (8.3)	18 (11.5)	0.03	23 (26.1)	11 (12.8)	34 (19.5)	<0.01
**2) Minimal pain**	17 (37)	34 (31.5)	51 (32.7)		37 (42.1)	24 (27.9)	61 (35.1)	
**3) Moderate pain**	16 (34.8)	49 (45.4)	65 (41.7)		24 (27.3)	34 (39.5)	58 (33.3)	
**4) Severe pain**	4 (8.7)	16 (14.8)	20 (12.8)		4 (4.6)	17 (19.8)	21 (12.1)	
**Overall satisfaction with pain management (1 = extremely dissatisfied, 10 = extremely satisfied); Median (IQR)**[2]	10 (8-10)	9 (7.5-10)	9 (8-10)	0.35	10 (8-10)	9 (7-10)	10 (8-10)	0.32

[1]One patient did not respond to pain score in the surgical extraction (opioid use) group
[2]Two patients did not respond to satisfaction in the surgical extraction group, one in opioid use and one in no opioid use

eTable 3. Sensitivity Analysis #2 (Excluding Patients Who Were Prescribed But Did Not Fill or Consume Opioid): Patient Outcomes Among Patients Who Were Prescribed and Consumed Opioid and Those Patients Who Were Not Prescribed an Opioid, Stratified by Procedure

	**Surgical Extraction**				**Routine Extraction**			
	**Non-Opioid Group N=46 (36.5)**	**Opioid Group N=80 (63.5)**	**Total N=126 (100)**	**P-value**	**Non-Opioid Group N=88 (56.4)**	**Opioid Group N=68 (43.6)**	**Total N=156 (100)**	**P-value**
**Outcomes.** Pain rating first week after dental procedure (n [%])[1]								
**1) No pain**	9 (19.6)	2 (2.5)	11 (8.8)	<0.01	23 (26.1)	6 (8.8)	29 (18.6)	<0.01
**2) Minimal pain**	17 (37)	26 (32.9)	43 (34.4)		37 (42.1)	18 (26.5)	55 (35.3)	
**3) Moderate pain**	16 (34.8)	35 (44.3)	51 (40.8)		24 (27.3)	29 (42.7)	53 (34.0)	
**4) Severe pain**	4 (8.7)	16 (20.3)	20 (16.0)		4 (4.6)	15 (22.1)	19 (12.2)	
**Overall satisfaction with pain management (1 = extremely dissatisfied, 10 = extremely satisfied); Median (IQR)[2]**	10 (8-10)	9 (8-10)	9 (8-10)	0.40	10 (8-10)	9 (7-10)	9.5 (8-10)	0.20

[1]One patient did not respond to pain score in the surgical extraction group
[2]Two patients did not respond to satisfaction in the surgical extraction group and two patients did not respond in the routine extraction group

©2020 Nalliah RP et al. *JAMA Network Open*
